# Dog Companionship and Loneliness in Community‐Dwelling Older Adults: A Multicentre, Cross‐Sectional Study

**DOI:** 10.1002/gps.70216

**Published:** 2026-05-02

**Authors:** Lauriane Segaux, Eric Poitrine, Marguerite Nicodème, Celia Herr, Anne‐Julie Vaillant‐Ciszewicz, Aline Hurtaud, Nolwenn Bombenger, Marie Boiteux‐Chabrier, Emeline Michel, Clémence Maurice, Caroline Gilbert, Florence Canoui‐Poitrine, Isabelle Fromantin

**Affiliations:** ^1^ Université Paris‐Est Créteil INSERM Institut Mondor de Recherche Biomédicale U955 Créteil France; ^2^ Maison de Santé Pluridisciplinaire Michael Balint Le Mée‐sur‐Seine France; ^3^ Département de Médecine Générale Sorbonne Université Paris France; ^4^ Nursing Department Wound Care and Research Unit Curie Institute Paris France; ^5^ Unités de Soins de Suite et de Réadaptation (SSR) Centre Hospitalier d’Avignon Avignon France; ^6^ CHU de Nice, Pôle Réhabilitation Autonomie VieillissementIRCAN Université Côte d'Azur (UNiCA) INSERM U1081 CNRS UMR 7284 Nice France; ^7^ Département universitaire de médecine générale, UFR médecine Université Reims Champagne‐Ardenne Reims France; ^8^ Assistance Publique Hôpitaux de Paris Hôpital Henri‐Mondor Unité de Recherche Clinique Créteil France; ^9^ Centre Hospitalier Universitaire de Nice Clinique Gériatrique et Thérapeutique Université Côte d'Azur Nice France; ^10^ Université Côte d'Azur LAMHESS Nice France; ^11^ Pôle Santé Samois‐sur‐Seine France; ^12^ Ecole Nationale Vétérinaire d'Alfort Maisons‐Alfort France; ^13^ Laboratoire Mecadev, UMR 7179 CNRS/MNHN Brunoy France; ^14^ Assistance Publique Hôpitaux de Paris Hôpital Henri‐Mondor Service de Santé Publique Créteil France

**Keywords:** community‐dwelling population, dog ownership, emotional well‐being, loneliness, older adults, social isolation

## Abstract

**Objectives:**

Loneliness and social isolation are major public health concerns among older adults and are associated with depression, cognitive decline, frailty, and loss of independence. Companion animals (and dogs in particular) might mitigate these adverse outcomes by fostering emotional support, physical activity, and social interactions. The objective of the present study aimed to evaluate the association between dog ownership and loneliness in community‐dwelling older adults.

**Methods:**

C‐KDOG is a multicentre, cross‐sectional study conducted between September 2020 and April 2023 at seven investigating centers in France. The participants were aged 75 or over and were living at home. Loneliness was assessed on the 11‐item De Jong Gierveld Loneliness Scale. The secondary outcomes included emotional and social loneliness subscores, social isolation (according to the Social Network Index). Associations between dog ownership and loneliness were analyzed using multivariable linear regressions adjusted for sociodemographic, environmental and clinical characteristics.

**Results:**

A total of 160 participants were included, of whom 47 were dog owners (mean age: 82 years; females: 116 (73%); living alone: 79 (49%)). The median overall loneliness scores did not differ significantly when comparing dog owners and non‐owners. In adjusted models, however, dog ownership was independently associated with lower loneliness. This association was mainly driven by a lower emotional loneliness subscore. Living alone, frailty, depressive symptoms, and sleep problems were independently associated with a greater level of loneliness. Dog ownership was primarily motivated by companionship (81%). Adverse events (such as falls or bites) were rare (5%).

**Conclusions:**

Dog ownership was associated with a lower level of emotional loneliness among community‐dwelling older adults, independently of living alone frailty and depression. Companion dogs might contribute to emotional well‐being in older adults. However, longitudinal studies are needed to confirm causality.

## Key Points

- Dog ownership was independently associated with lower levels of loneliness in community‐dwelling older adults, with a particularly strong effect on emotional loneliness.
- Living alone, frailty, depressive symptoms, and sleep problems were significant factors associated with higher loneliness, regardless of pet ownership.
- Most older adults owned a dog for companionship, and adverse events related to dog ownership (falls or bites) were uncommon

## 1 Introduction

Approximately one in three adults reports frequent feelings of loneliness, and about one in four older adults experiences social isolation [1]. Although these two concepts are distinct (loneliness corresponds to a feeling of distress resulting from perceived social isolation, whereas isolation corresponds to a lack of social interactions), their consequences are intertwined. Older adults experiencing social isolation frequently report higher levels of depression, anxiety and cognitive decline and frailty and multimorbidity [2, 3]. Loneliness is not merely a representation of emotional discomfort; it is increasingly recognized as an independent risk factor for poor physical and mental health in older adults [4].

Loneliness significantly increases the risk of depression, exacerbates depressive symptoms, and contributes to depression onset independently of other risk factors [5]. The literature data consistently show that loneliness is a significant predictor of anxiety disorders, which further highlights the importance of social connectedness for psychological health [6, 7]. Furthermore, loneliness has been associated with accelerated cognitive decline and an elevated dementia risk; hence, it is a public health issue in aging populations [8]. Addressing loneliness is therefore of relevance for preventive strategies and public health interventions targeting older adults [9]. Literature distinguishes two types of loneliness: emotional loneliness, arising from the absence of close, intimate attachment relationships, and social loneliness, which results from a lack of a broader social network or sense of social integration [10]. This multidimensional conceptualization is operationalized in the De Jong Gierveld Loneliness Scale, which explicitly measures emotional and social loneliness as related but distinct components of the loneliness experience [11].

Frailty is another concern exacerbated by loneliness and isolation among older adults. It is characterized by diminished physiological reserves and results in a greater incidence of adverse health events, such as falls, hospital admission, and loss of independence [12]. Prolonged social isolation reduces opportunities for meaningful social engagement and physical activities, which accelerates the onset of frailty. The interplay between physical frailty and social isolation creates a vicious cycle in which frailty can lead to greater isolation (due to reduced mobility), and isolation can further accelerate frailty (through diminished physical activity and a lack of social stimulation) [13, 14]. Breaking this cycle is important for promoting the health, independence, and well‐being of older adults.

Companion animals in general and dogs in particular have emerged as promising interventions for addressing these interconnected challenges and mitigating the negative impacts of social isolation and loneliness among older adults [15, 16]. Uniquely, dogs provide consistent and authentic companionship, emotional support, and daily routines that involve regular feeding, grooming and exercise (such as walking). The structured daily routines associated with dog ownership offer many benefits for physical health. Regular physical activities like dog walking have been shown to improve mobility, decrease the risk of frailty, and enhance physical fitness among older adults [14, 17, 18, 19, 20]. Furthermore, dogs may facilitate social interactions through casual exchanges during neighborhood walks or visits to parks [21]; these may create opportunities for new friendships and community connections and thereby alleviating subjective feelings of loneliness [22].

Beyond the promotion of physical health and social engagement, dog companionship significantly increases psychological resilience in older adults [23]. Dogs provide continuous emotional support by offering a reliable presence that helps older adults to cope with life's changes, including bereavements and post‐retirement transitions in social roles [24]. Research indicates that older adults who own dogs report lower levels of anxiety and depression ‐ demonstrating the psychological benefits of pet companionship [25]. Furthermore, regular interactions with dogs can help mitigate stress responses, enhance emotional stability, and offer a sense of purpose and structure that is often missing from isolated older adults' daily lives [26]. Consequently, dogs can act as crucial sources of emotional support by helping older adults to adapt to life transitions and cope with the emotional impacts of aging.

Despite having these notable benefits, dog ownership also carries potential risks and challenges ‐ particularly for older adults. Mobility limitations, financial constraints, and the physical demands associated with pet care can pose significant barriers and potential hazards. The responsibility of owning a dog (such as routine care tasks and regular visits to the vet) can present substantial burdens for older adults in general and those with declining mobility or financial limitations in particular. Furthermore, pet‐related accidents (particularly falls) are a noteworthy risk factor for injuries among older adults [27]. These considerations highlight the importance of well‐thought‐out strategies for maximizing the positive impacts of dog ownership while minimizing the associated risks. Targeted interventions (such as dog obedience training [28], community support programs providing pet care assistance, and financial support for veterinary expenses) can help older adults to safely and successfully integrate dog companionship into their lives.

Given the potential benefits and challenges associated with dog ownership, the primary objective of the present study was to explore the relationship between having a companion dog at home and loneliness among older adults. The secondary objectives were to investigate associations between dog ownership on one hand and social and emotional loneliness, social isolation, and the risks of falls and bites on the other.

## 2 Methods

### 2.1 Design, Setting and Participants

C‐KDOG is a multicentre, cross‐sectional, comparative study of people aged 75 and over living at home. Patients were included between September 2020 and April 2023 in one of seven investigating centers in mainland France (three hospitals and four primary care settings). Participants were eligible if they were aged 75 years or older and living at home (including living with relatives or friends, or in senior residences or sheltered housing), but not in nursing homes or long‐term care units. Exclusion criteria comprised being bedridden, living in a nursing home, long‐term care unit, or without stable housing, owning a pet other than a dog, having experienced the loss of a dog within the 12 months prior to inclusion, presenting insufficient French language comprehension to validly complete questionnaires, having cognitive, mental, or psychiatric disorders precluding protocol participation, or being under legal guardianship.

### 2.2 Ethical Approval and Consent to Participate

The protocol was approved by an independent ethics committee (*Comité de Protection des Personnes Est I*, Dijon, France; reference: SI 19.07.29.61314).

Participants received both verbal and written information about the study's objectives and procedures. Verbal consent was obtained by the investigator prior to inclusion.

### 2.3 Data Collection

Data were collected prospectively. The assessment (an interview and a clinical examination) was carried out by an investigator (a nurse or a physician). The investigators attended an introductory session on the use of the validated assessment tools.

Five to 10 days after the inclusion visit, the patient was contacted by phone by a clinical study assistant, in order to collect additional information (place of residence, household composition, educational level, employment).

The dog owners were interviewed a second time, to gather information on the animal. This second interview was conducted by a dog trainer. Data were standardized and collected in an electronic case report form (CleanWeb, Telemedicine Technologies SAS, Boulogne‐Billancourt, France).

### 2.4 Endpoint

The primary endpoint was the level of loneliness, as self‐assessed on the De Jong Gierveld Loneliness 11‐item Scale [11]; this instrument has been widely validated (including as a French‐language version) and was designed to measure emotional and social dimensions of loneliness [11]. The scale consists of negatively worded and positively worded statements, to which respondents indicate their degree of agreement on a three‐point scale (“yes”, “more or less”, and “no”). The higher the total score, the greater the level of loneliness. An emotional loneliness subscore is derived from the items focusing on the absence of intimate relationships, whereas a social loneliness subscore is derived through items addressing a lack of broader social integration. (Appendix 1)

The secondary endpoints were the social loneliness subscore, the emotional subscore, and the social isolation. Social isolation was self‐assessed via the 4‐item Social Network Index including the following questions: “Do you share your life with someone living in the same household as you?”, “Do you belong to a social group?”, “How many people do you trust and can rely on?” And “How many direct contacts per month do you have with your family or friends, at your home or theirs?” (Appendix 2).

### 2.5 Covariates

Sociodemographic and clinical data were collected. Sociodemographic variables included sex, age, living environment (personal home, with family member/third party or Residential home/residence for older adults) and distance to the nearest green space (5 options ranging from a garden to more than 1 km), educational level and former profession. Educational level was assessed by asking participants to report their highest qualification obtained according to the French educational level and translated into the following categories: Primary school certificate, GCSEs under grade C, NVQ, BETC or GNVQ, BETC Higher National Diploma, BA or BS, MA or MS, Other, No degree, and Don't know. Due to sample size considerations, the BETC and BA or BS categories were grouped together, as were the No degree and Don't know categories. Former profession was assessed using the following categories: Farmers, Craftsmen, shopkeepers and company directors; Executives and higher intellectual professions; Intermediate professions; Employees; Workers; Not working or at home; and Other.

Clinical variables included the number of comorbidities, the number of medications taken daily, cognitive function as assessed by a clinician. Patients were asked whether they had a primary carer (yes/no), current anxiety disorder (yes/no), problems sleeping (never, sometimes, often, very often, constantly), due to sample size, the “very often” and “constantly” categories were combined.

History of falls within the previous six months was assessed using a dichotomous question (yes/no). In the case of a positive response, further information was collected regarding falls with complications (yes/no), post‐fall syndrome (yes/no), the number of falls and whether the falls were caused by the dog (yes/no).

Independence was assessed with regard to activities of daily living (ADL) and instrumental activities of daily living (IADL). The Cumulative Illness Rating Scale‐Geriatric was used to measure comorbidities. Frailty was assessed using the Short Emergency Geriatric Assessment Score (SEGA), encompassing 13 items: age, living environment, medications, mood, health perception, falls in the past 6 months, nutrition, comorbidities, mobility, continence, cognitive function, IADL, and meal management. Each item is scored from 0 (the most favorable state) to 2 (the least favorable). Patients were considered to be “not very frail” if their score was ≤ 8, “frail” if the score was between 8 and 11, and “very frail” if the score was > 11.

The mini–Geriatric Depression Scale was used to screen for depression; patients with at least one abnormal response were considered to be at risk of depression.

The Marshall self‐questionnaire was used for a brief assessment of physical activity; the score ranges from 0 to 8, and a score of 0–3 indicates that the person does not perform sufficient physical activity.

Information about the animal's age and sex was requested and the following questions were also asked to dog owners: Do you usually walk your dog outdoors (yes/no)? How often do you walk your dog (a few times a week, once a day, twice a day, more than twice a day)? On a scale of 0–10, would you say that your dog is an emotional support (0: absolutely not, 10: you cannot do without him). Length of relationship with the animal, and reason for getting a dog were also asked, with the following categories (yes/no): security/guard, hunting, companionship, going out/doing sport, assistance (hearing, sight), other, how does your dog behave around other dogs or people: solitary/sociable/aggressive. Finally, subjects were asked whether they felt they had expanded their social circle thanks to their dog (yes/no).

Individuals who did not have a pet were asked to select one of the following reasons (yes/no): health condition, fear of animals, financial cost, too old (fear of dying before the animal), too many responsibilities, suffering due to the death of a previous pet, refusal imposed by your housing provider, unsuitable housing, distance from a green space, dislike of dogs, no interest in pets, refusal by spouse (if applicable), other constraints.

The possible accidents (bites, falls, or home degradation) caused by the animal, were also asked of the participants.

### 2.6 Statistical Analyses

Data were expressed as the frequency (percentage) or the median [interquartile range (IQR)], as appropriate.

The characteristics of the dog owners versus the non‐owners were compared using Pearson's chi‐squared test or Fisher's exact test for qualitative variables and Student's *t* test or the Wilcoxon‐Mann‐Whitney test for quantitative variables.

The association between dog ownership and the level of loneliness was analyzed using linear regression. The assumptions for applying linear regression in a valid manner were checked. Coefficients were reported with their 95% confidence intervals (Cis). The associations between others potential determinants (sociodemographic, clinical, and environmental factors) were also assessed. For the multivariable regression model, variables with *p* < 0.2 in a univariate analysis and known or suspected loneliness determinants (such as age, sex, inclusion center (related to place of residence), depression and independence [29, 30, 31]) were tested.

We looked for interactions between variables suspected of being associated with (or modifying the association with) our outcomes. Potential interaction terms were tested based on a priori hypotheses: to this regard, we looked whereas the link between variables having a dog and our outcomes was modified by GDS score, living alone and the admission center. Only statistically significant interactions were retained in the final models. Due to the presence of missing data, multiple imputations were carried out under the “missing at random” hypothesis. Missing values in the SEGA score, the Geriatric Depression Scale short‐form, and distance from a green space were handled using multiple imputation by chained equations (MICE) with logistic regression.

The threshold for statistical significance was set to *p* < 0.05.

All analyses were carried out with Stata software (version 17.0, StataCorp, College Station, TX, USA).

## 3 Results

A total of 160 patients (including 47 dog owners) were included by the seven centers. In the study population as a whole, the mean age was 82 years, 73% of the participants were female, and 49% lived alone (Appendix 3).

The dog owners were slightly younger, less likely to be physically active, and more likely to have a home with a garden, to be polymedicated, to have problems sleeping, and to be dependent for instrumental activities of daily living (Appendix 3).

Two‐thirds of the dog owners reported walking their dogs from a few times a week to more than twice a day. Most of the non‐walkers (92%) had a garden. The median age of the dog was 6 years, and 51.4% of the dogs were female (Appendix 4). The median duration of the relationship between the owner and the dog was 4 years. The reasons cited for owning a dog were companionship (81%), being offered the dog or having adopted it (49%), the desire to go out or do more physical activity (35%), and hunting (3%). The reasons for not owning a dog included responsibilities that were deemed to be too great (60%), old age (25%), other constraints (such as frequent travel) (21%), and unsuitable accommodation (16%). Among the 47 dog owners, the median [IQR] score (on a 0–10 scale) for the emotional support provided by their pet was 10 [9–10]. Approximately 75% of the dog owners reported that their dog was sociable toward other dogs and other people. More than a third of the owners reported that thanks to their dog, their social circle had expanded. Incidents involving a fall caused by the dog or being bitten were reported by 5% of dog owners (Appendix 4).

**FIGURE 1:**
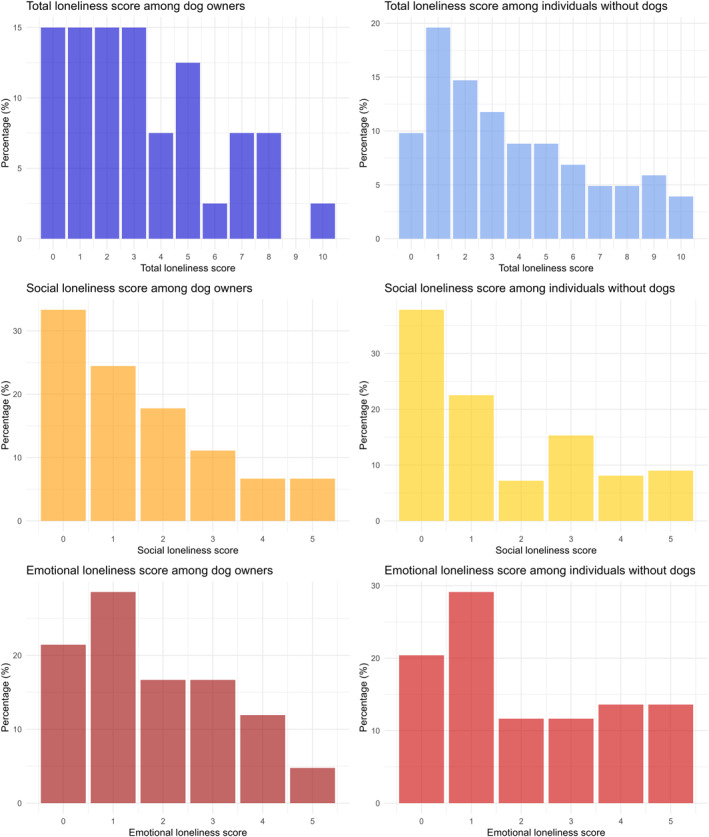
Histograms of the total score on the 11‐item De Jong Gierveld Loneliness Scale and the social and emotional subscores, as a function of dog ownership. The higher the score, the greater the feeling of loneliness.

The median total loneliness score was 3 out of 11 overall and was similar in dog owners and non‐owners (Table 1 and Figure 1). The median social loneliness score was 1 out of 5, and the median emotional loneliness score was 2 out of 6; again, there were no significant differences in these scores between dog owners and non‐owners. Similarly, the proportion of individuals not belonging to a social group or having fewer than eight trusted persons did not depend on dog ownership. In terms of social contacts, there was a nonsignificant trend toward more regular visits among dog owners (55.3%, vs. 41.6% among non‐owners; *p* = 0.153) (Table 1).

**TABLE 1:** Univariates analyses of the association between dog ownership and social isolation and the total loneliness score or the social and emotional loneliness scores.

		Dog’ s ownership		
	Total	No	Yes	*p*‐value^a^
	*N* (%)	*N* (%)	*N* (%)	
	*N* = 160	*N* = 113	*N* = 47	
Total loneliness score/11, median [interquartile range](MD = 18/11.3%)	3.00 [1.00–6.00]	3.00 [1.00–6.00]	3.00 [1.00–5.00]	0.632
Social loneliness subscore/5 (MD = 4/2.5%)	1.00 [0.00–3.00]	1.00 [0.00–3.00]	1.00 [0.00–2.00]	0.968
Emotional loneliness subscore/6 (MD = 15/9.4%)	2.00 [1.00–4.00]	2.00 [1.00–4.00]	2.00 [1.00–3.00]	0.495
Social network index				
Does not belong to a particular social group	108 (67.92)	76 (67.26)	32 (69.57)	0.777
Less than 8 people you can trust and rely on	118 (73.75)	83 (73.45)	35 (74.47)	0.894
Number of direct contacts per month with family, friends, at home or in their homes				0.153
Very few or none	32 (20.00)	22 (19.47)	10 (21.28)	
Moderate visits	55 (34.38)	44 (38.94)	11 (23.40)	
Regular visits	73 (45.63)	47 (41.59)	26 (55.32)	

*Note:* (/%) indicates the number and % of missing values.

Abbreviation: MD, missing data.

^a^ *p* value in a Mann‐Whitney or chi‐squared test, as appropriate.

The total, social and emotional loneliness scores were higher among (i) people with anxiety and depressive symptoms, (ii) people in average or poor general condition (compared with those in very good general condition), and (iii) people who were frail or very frail. The total and emotional loneliness scores were lower among people who share their lives with someone living in the same home and the three scores were lower among people who had eight or more people they trust and rely on (Table 2).

**TABLE 2:** Univariate analyses of the association between loneliness scores and participant characteristics.

	Total loneliness score *N* = 142		Social loneliness subscore *N* = 156		Emotional loneliness subscore *N* = 145	
	Beta^a^ [CI 95%]	*p*‐value^a^	Beta^a^ [CI 95%]	*p*‐value^a^	Beta^a^ [CI 95%]	*p*‐value^a^
Age, per 12‐month increment	0.02 [−0.07–0.10]	0.699	0.04 [−0.01–0.08]	0.133	−0.02 [−0.07–0.03]	0.351
Female (vs. male)	0.18 [−0.89–1.25]	0.740	−0.22 [−0.81–0.36]	0.455	0.17 [−0.45–0.79]	0.591
Educational level						
No degree/Don't know	0 [ref]	0.829	0 [ref]	0.839	0 [ref]	0.905
Primary school certificate	0.39 [−1.92–2.71]	0.736	−0.28 [−1.58–1.02]	0.673	0.67 [−0.66–1.99]	0.321
GCSEs under grade C	−0.17 [−2.23–1.88]	0.868	−0.32 [−1.46–0.81]	0.574	0.17 [−1.01–1.34]	0.779
NVQ, BETC or GNVQ	0.13 [−1.62–1.89]	0.881	−0.19 [−1.19–0.80]	0.698	0.36 [−0.64–1.36]	0.475
BETC higher national diploma, BA or BS	−0.35 [−2.34–1.64]	0.726	−0.56 [−1.68–0.56]	0.325	0.17 [−0.95–1.29]	0.769
MA or MS	0.56 [−1.50–2.62]	0.590	0.11 [−1.08–1.30]	0.855	0.60 [−0.55–1.76]	0.302
Other	−1.12 [−3.31–1.07]	0.312	−0.69 [−1.88–0.50]	0.253	0.00 [−1.25–1.25]	1.000
Former profession						
Craftsmen, shopkeepers, company directors	0 [ref]	**0.014**	0 [ref]	0.309	0 [ref]	**0.009**
Executives and higher intellectual professions	0.72 [−1.68–3.11]	0.555	−0.19 [−1.48–1.09]	0.766	1.06 [−0.30–2.43]	0.126
Intermediate professions	2.07 [−0.27–4.42]	0.083	0.49 [−0.80–1.78]	0.454	1.43 [0.08–2.79]	**0.038**
Employees	2.12 [0.00–4.25]	0.050	0.21 [−0.95–1.37]	0.718	1.73 [0.51–2.96]	**0.006**
Workers	5.75 [2.40–9.10]	**0.001**	1.95 [0.00–3.90]	0.050	3.63 [1.69–5.56]	**< 0.001**
Not working or at home	0.88 [−1.86–3.61]	0.528	−0.19 [−1.71–1.33]	0.806	0.87 [−0.70–2.45]	0.274
Other	2.48 [0.27–4.69]	**0.028**	0.47 [−0.76–1.70]	0.451	1.70 [0.43–2.96]	**0.009**
Inclusion date						
2020	0 [ref]	0.392	0 [ref]	0.154	0 [ref]	0.636
2021	0.05 [−1.60–1.70]	0.953	0.28 [−0.61–1.16]	0.537	−0.28 [−1.25–0.68]	0.561
2022	−0.85 [−2.37–0.67]	0.270	−0.36 [−1.17–0.46]	0.389	−0.48 [−1.37–0.41]	0.289
2023	−0.31 [−2.27–1.65]	0.756	0.35 [−0.72–1.42]	0.519	−0.66 [−1.82–0.49]	0.259
Inclusion at a hospital versus a primary care center	−0.75 [−1.75–0.25]	0.142	−0.32 [−0.87–0.24]	0.259	−0.44 [−1.02–0.14]	0.139
Owning a dog	−0.36 [−1.41–0.70]	0.505	−0.07 [−0.65–0.51]	0.811	−0.26 [−0.87–0.34]	0.390
Living alone	1.47 [0.55–2.39]	**0.002**	0.51 [−0.01–1.03]	0.053	0.79 [0.25–1.32]	**0.004**
Living space						
Personal home	0 [ref]	0.711	0 [ref]	0.940	0 [ref]	0.451
With a family member or third party	−0.64 [−6.45–5.16]	0.827	−0.56 [−3.90–2.77]	0.739	−0.08 [−3.44–3.28]	0.960
Residential home/residence for older adults	1.36 [−2.02–4.74]	0.428	0.10 [−1.84–2.05]	0.916	1.25 [−0.71–3.20]	0.209
Distance from a green space						
At home (garden)	0 [ref]	**0.011**	0 [ref]	**0.009**	0 [ref]	**0.048**
Between 0 and 200 m	1.07 [−0.16–2.31]	0.088	0.26 [−0.40–0.92]	0.439	0.83 [0.11–1.55]	**0.024**
Between 200 and 500 m	2.44 [0.92–3.97]	**0.002**	1.38 [0.52–2.24]	**0.002**	1.03 [0.13–1.92]	**0.025**
More than 500m	1.51 [−0.04–3.07]	0.057	0.88 [0.02–1.74]	**0.046**	0.65 [−0.26–1.56]	0.159
Current smoking						
Non‐smoker	0 [ref]	0.299	0 [ref]	0.400	0 [ref]	0.293
Active smoker	1.38 [−0.41–3.17]	0.130	0.61 [−0.38–1.60]	0.225	0.80 [−0.21–1.81]	0.118
Former smoker	0.34 [−0.83–1.50]	0.569	0.25 [−0.38–0.88]	0.432	0.11 [−0.56–0.79]	0.739
Current general condition						
Very good	0 [ref]	**0.003**	0 [ref]	0.050	0 [ref]	**0.002**
Good	0.74 [−0.36–1.84]	0.183	0.27 [−0.36–0.89]	0.396	0.56 [−0.08–1.20]	0.087
Fair	2.32 [0.98–3.66]	**0.001**	0.94 [0.17–1.71]	**0.017**	1.46 [0.67–2.25]	**< 0.001**
BMI	−0.02 [−0.13–0.09]	0.687	0.00 [−0.06–0.06]	0.905	−0.02 [−0.08–0.05]	0.611
Number of medications taken daily	0.17 [0.01–0.33]	**0.037**	0.12 [0.02–0.21]	**0.014**	0.07 [−0.02–0.16]	0.148
Number of illnesses requiring regular treatment	0.24 [−0.12–0.60]	0.196	0.15 [−0.05–0.35]	0.150	0.11 [−0.10–0.32]	0.290
Overall CIRS‐G score	0.09 [−0.04–0.23]	0.178	0.09 [0.02–0.17]	**0.017**	0.00 [−0.07–0.08]	0.913
IADL score	0.09 [−0.24–0.42]	0.596	−0.02 [−0.20–0.16]	0.835	0.07 [−0.11–0.26]	0.450
ADL score	−0.32 [−1.32–0.67]	0.522	−0.24 [−0.80–0.32]	0.400	−0.05 [−0.63–0.54]	0.876
Marshall score	−0.13 [−0.35–0.08]	0.215	−0.09 [−0.21–0.03]	0.131	−0.06 [−0.19–0.06]	0.315
Frail/very frail person	1.92 [0.89–2.94]	**< 0.001**	0.95 [0.37–1.52]	**0.001**	0.92 [0.31–1.52]	**0.003**
Has a main carer	−0.55 [−1.68–0.57]	0.331	−0.26 [−0.88–0.35]	0.398	−0.20 [−0.84–0.44]	0.538
No daily physical activity	0.42 [−0.66–1.49]	0.445	0.62 [0.04–1.21]	**0.037**	−0.20 [−0.83–0.42]	0.517
Number of minutes walked per day	−0.01 [−0.03–0.02]	0.550	−0.01 [−0.02–0.00]	0.169	0.00 [−0.01–0.01]	0.948
Number of falls	−0.18 [−0.92–0.56]	0.629	0.17 [−0.08–0.41]	0.175	−0.32 [−0.75–0.12]	0.151
History of falls in the last 6 months	0.08 [−0.93–1.08]	0.880	0.16 [−0.40–0.71]	0.571	−0.04 [−0.62–0.53]	0.881
Falls with complications	−0.96 [−2.75–0.84]	0.288	−0.31 [−1.33–0.71]	0.546	−0.87 [−1.95–0.21]	0.110
Post‐fall syndrome	1.18 [−0.93–3.30]	0.266	1.72 [0.67–2.77]	**0.002**	−0.43 [−1.71–0.86]	0.506
Slightly impaired cognitive function	1.03 [−0.35–2.42]	0.141	0.53 [−0.19–1.25]	0.149	0.38 [−0.44–1.19]	0.362
Abnormal GDS score (short‐form)	2.83 [1.99–3.68]	**< 0.001**	1.18 [0.68–1.69]	**< 0.001**	1.63 [1.14–2.12]	**< 0.001**
Current anxiety disorder	2.17 [1.27–3.07]	**< 0.001**	0.89 [0.37–1.40]	**0.001**	1.12 [0.60–1.65]	**< 0.001**
Current depressive syndrome	2.53 [1.52–3.55]	**< 0.001**	1.00 [0.42–1.58]	**0.001**	1.38 [0.78–1.97]	**< 0.001**
Fatigue						
Never	0 [ref]	0.052	0 [ref]	0.104	0 [ref]	**0.026**
Sometimes	0.36 [−0.90–1.62]	0.571	−0.14 [−0.84–0.55]	0.684	0.38 [−0.35–1.11]	0.306
Often	−0.63 [−2.18–0.91]	0.420	−0.87 [−1.73 to −0.01]	**0.048**	0.23 [−0.67–1.13]	0.613
Very often/constantly	1.41 [−0.01–2.84]	0.051	0.16 [−0.64–0.96]	0.696	1.18 [0.37–2.00]	**0.005**
Sleep problems						
Never	0 [ref]	**0.002**	0 [ref]	0.124	0 [ref]	**0.000**
Sometimes	0.48 [−0.68–1.63]	0.415	0.01 [−0.65–0.66]	0.980	0.49 [−0.17–1.15]	0.141
Often	1.99 [0.57–3.42]	**0.006**	0.82 [−0.01–1.64]	0.052	1.05 [0.22–1.88]	**0.013**
Very often/constantly	2.20 [0.87–3.54]	**0.001**	0.50 [−0.25–1.25]	0.190	1.62 [0.85–2.39]	**0.000**
Social network index score	−1.12 [−1.46–0.78]	**< 0.001**	−0.66 [−0.85 to −0.48]	**< 0.001**	−0.37 [−0.58 to −0.15]	**0.001**
Does not belong to a particular social group	0.46 [−0.56–1.48]	0.374	0.39 [−0.17–0.96]	0.167	−0.01 [−0.60–0.58]	0.966
Less than 8 people you can trust and rely on	1.99 [0.96–3.02]	**< 0.001**	1.19 [0.63–1.75]	**< 0.001**	0.72 [0.10–1.34]	**0.024**
Number of direct contacts per month with family, friends, at home or in their homes						
Regular visits	0 [ref]	**< 0.001**	0 [ref]	**< 0.001**	0 [ref]	0.149
Moderate visits	0.92 [−0.10–1.94]	0.076	0.70 [0.17–1.23]	**0.009**	0.29 [−0.33–0.92]	0.354
Very few or none	2.75 [1.61–3.89]	**< 0.001**	2.04 [1.42–2.66]	**< 0.001**	0.70 [−0.01–1.41]	0.053

*Note:* The bold values indicate statistically significant tests (*p*‐value < 0.05).

Abbreviations: ADL, activities of daily living; BA, Bachelor of Arts; BS, Bachelor of Science; BTEC, Business and Technology Education Council; BMI, body mass index; CIRS‐G, Cumulative Illness Rating Scale‐Geriatric, GCSE, General Certificate of Secondary Education; GDS, Geriatric Depression Scale; GNVQ, General National Vocational Qualification; IADL, Instrumental activities of daily living; MA, Master of Arts; MS, Master of Science; NVQ, National Vocational Qualification.

^a^ Estimated coefficients and the *p*‐value derived from an univariate linear regression model. *β* coefficients indicate the mean change in total loneliness score per unit increase in each predictor, for example, living alone was associated with a 1.47‐point greater total loneliness score, compared with not living alone.

Individuals who were visited regularly by family members or friends had lower total and social loneliness scores than those who had very few or no visits. In univariate analyses, the year of inclusion (compared with 2020), the ADL score, the IADL score, cognitive function, number of falls, and dog ownership were not associated with the total loneliness score or the social or emotional loneliness subscores (Table 2).

In a multivariable analysis of the total loneliness score, dog ownership (Beta [CI 95%]: −1.20 [−2.21–0.18]) by older adults was independently associated with a lower level of loneliness. In contrast, living alone, frailty, depressive symptoms, frequent sleep problems were independently associated with greater total and emotional loneliness and few or no social contacts were independently associated with greater total and social loneliness. Being included by one of two study centers in a wealthier urban area was associated with a lower level of total and emotional loneliness, compared with inclusion by a reference center based in a semirural, deprived, primary care setting (Table 3).

**TABLE 3:** Multivariable analyses of the association between loneliness scores and participant characteristics.

	Total loneliness score		Social loneliness score		Emotional loneliness score	
	*N* = 139		*N* = 153		*N* = 142	
	Beta (CI 95%)	Adjusted *p*‐value^a^	Beta (CI 95%)	Adjusted *p*‐value^a^	Beta (CI 95%)	Adjusted *p*‐value^a^
Age	−0.05 [−0.12–0.03]	0.232	0.00 [−0.048–0.05]	0.970	−0.05 [−0.10 to −0.01]	**0.023**
Dog ownership	−1.20 [−2.21–0.18]	**0.022**	−0.26 [−0.88–0.35]	0.397	−0.80 [−1.40 to −0.20]	**0.010**
Living alone	1.24 [0.45–2.02]	**0.002**	0.47 [−0.14–0.96]	0.057	0.52 [0.05–1.00]	**0.030**
Does not have a garden	0.42 [−0.53–1.36]	0.386	0.27 [−0.31–0.85]	0.352	0.22 [−0.32–0.76]	0.428
Frail/very frail person	1.05 [0.09–2.01]	**0.033**	0.25 [−0.36–0.87]	0.418	0.71 [0.13–1.28]	**0.016**
Abnormal GDS score (short‐form)	1.49 [0.61–2.37]	**0.001**	0.43 [−0.11–0.97]	0.119	1.16 [0.63–1.69]	**< 0.001**
Sleep problems						
Never	0		0		0	
Sometimes	0.50 [−0.50–1.50]	0.325	0.00 [−0.62–0.62]	0.996	0.50 [−0.10–1.10]	0.105
Often	1.02 [−0.19–2.24]	0.099	0.43 [−0.34–1.20]	0.271	0.41 [−0.33–1.14]	0.277
Very often/constantly	2.15 [1.00–3.29]	**< 0.001**	0.57 [−0.13–1.26]	0.109	1.41 [0.71–2.10]	**< 0.001**
Less than 8 people of trust	0.96 [0.03–1.88]	**0.042**	0.52 [0.06–1.10]	0.076	0.35 [−0.21–0.91]	0.219
Social network index						
Number of direct contacts per month with family/friends, at home/in their homes						
Regular visits	0		0		0	
Moderate visits	0.65 [−0.28–1.58]	0.167	0.37 [−0.21–0.94]	0.210	0.23 [−0.32–0.78]	0.402
Very few or none	1.58 [0.47–2.69]	**0.006**	1.40 [0.69–2.10]	**< 0.001**	0.11 [−0.56–0.78]	0.744
Center number^b^						
1	0		0		0	
2	−1.89 [−3.22–0.77]	**0.001**	−0.65 [−1.34–0.04]	0.064	−1.09 [−1.75 to −0.42]	**0.002**
4	−1.12 [−2.55–0.30]	0.121	0.39 [−0.49–1.28]	0.380	−1.32 [−2.18 to −0.46]	**0.003**
5	−1.15 [−2.86–0.57]	0.188	0.01 [−1.00–1.02]	0.984	−0.70 [−1.70 to −0.30]	0.166
6	−1.62 [−4.29–1.04]	0.230	0.41 [−1.31–2.14]	0.636	−2.03 [−3.64 to −0.42]	**0.014**
8	−1.73 [−2.92–0.53]	**0.005**	−0.37 [−1.12–0.37]	0.322	−1.20 [−1.92 to −0.48]	**0.001**
9	−2.70 [−5.46–0.06]	0.055	−0.92 [−2.70–0.86]	0.309	−1.65 [−3.31–0.02]	0.053

*Note:* The bold values indicate statistically significant tests (*p*‐value < 0.05).

Abbreviation: GDS, Geriatric Depression Scale.

^a^ Reported *p*‐values correspond to adjusted values from the linear regression model, after accounting for all the variables included in the model. For example, dog ownership was associated with a 1.20‐point lower total loneliness score, after adjustment for all the variables in the model.

^b^ Centers 3 and 7 were enrolled as investigating centers but did not include any participants.

With regard to the social loneliness score, most associations were not statistically significant. However, having very few or no direct contacts was associated with greater loneliness (Table 3).

Dog ownership was independently associated with a lower level of emotional loneliness (−0.80 [−1.40 to −0.20]). Younger age, living alone, frailty, depressive symptoms, and frequent sleep problems were independently associated with greater loneliness. Several study centers also showed significantly lower emotional loneliness subscores, compared with the reference center in a primary care setting.

## 4 Discussion

Our survey results highlighted a positive association between dog ownership and a lower degree of loneliness in older patients in hospital and in primary care settings. The association was independent of the main confounding factors, such as social isolation, frailty, and mood. The association was driven by a lower emotional loneliness subscore, rather than the social loneliness subscore. Dog owners stated that they had decided freely to have a dog and wanted companionship and that dog ownership was a driver for social and physical activity. Moreover, all the dog owners scored the emotional support provided by the animal at 10 out of 10. Non‐owners stated that ownership involved too much responsibility and were afraid of dying before the dog.

An association between dog ownership and a lower level of loneliness was also found in two cross‐sectional studies conducted in Germany. One investigated 63 dog owners aged 65 and over, and the other investigated 66 dog owners aged 80 and over. Both studies found that dog owners reported a lower level of loneliness than other pet owners and non‐pet owners [32, 33].

These findings emphasize the strong and positive relationship between dogs and older adults. In our study, the owners stated that their dog was sociable with other dogs and other humans; this is in line with the results of a study conducted in England and that tested the dog's propensity to initiate interaction with passers‐by [34]. Regarding sociability mediated by the dog, the owners seemed less categorical, with only one third having been able to expand their social network thanks to the dog. Ye, a recent, qualitative study conducted in Australia identified social inclusion and participation as one of the four pillars of a companion dog's role for an older person [35].

Stimulation for physical activity was the third motivating factor for dog ownership in our sample. Indeed, the dog owners reported less physical activity than non‐owners but more walking time. Two‐thirds of the dog owners reported walking their dogs, although the most of the non‐walkers had a garden. Indeed, studies of dog‐owning older adults have found that the dog promotes physical activity, and that owners who walk their dogs have better social functioning and emotional well‐being [23, 36]. These findings suggest that walking a dog may mediate the interaction between dog ownership and better social functioning and emotional well‐being. In our study, the effect of the dog's presence on the feeling of loneliness appeared to be linked to the relationship between the owner and their pet as much as it was to the potential for new encounters during walks. This observation is consistent with that of Hui Gan et al. with regard to the other benefits of companion dogs for the owner: they provide a presence, security, structure, and a meaningful role [35]. The results of a Spanish study of more severely dependent older people also found an association with a lower level of loneliness, which was partly independent of outings with the animal [15]. The latter study also highlighted the importance of matching the type of animal to the owner's functional capacity. In our study, most of the dog owners mostly report owning small dogs, such as terriers and toy dogs. This reflects a form of common sense among older people, in which they take account of both the animal's well‐being and their own well‐being. Moreover, only 5% of the dog owners in our study reported having a fall caused by the dog or having been bitten; this results is in line with the literature data [37].

Our univariate analyses did not evidence a significant association between dog ownership and loneliness. However, after adjustment for sociodemographic, environmental and clinical variables, dog ownership became significantly associated with a lower level of loneliness. This change might be due (at least in part) to the confounding role of several factors related to both dog ownership and loneliness. For instance, individuals living in houses with gardens are more likely to own a dog; access to private green space is independently associated with a lower level of loneliness and improved social well‐being [38, 39]. Similarly, dog ownership is more prevalent in semirural areas and small towns than in large urban centers (such as our investigating centers 2 and 8), where housing constraints and lifestyles are less compatible with pets. In parallel, urban residents in large metropolitan areas report higher levels of loneliness [40]. Sleep problems might also confound the association: some individuals with insomnia or poor sleep quality may seek pets for emotional comfort, while loneliness itself has been shown to be linked to impaired sleep [41]. Taken as a whole, these findings suggest that adjustment for confounding factors provides a clearer understanding of the extent to which dog ownership is independently associated with a lower level of loneliness.

Our study had several strengths. Firstly, we characterized the patients' clinical and sociodemographic characteristics in depth; this enabled us to take account of social and clinical factors that might have confounded our assessment of the potential link between dog ownership and loneliness on its emotional side. To our knowledge, these elements have not been reported in previous studies. The findings suggest the existence of a specific bond between dogs and older adults. This result is consistent with Scheibeck's qualitative study describing the animal as a source of emotional enrichment. That study highlights strong empathic experiences with the dog and grief after its loss comparable to that felt for a relative or close friend [42]. Secondly, we have clearly distinguished between loneliness and social isolation. Lastly, our survey was designed to characterize the respondents' motivations for owning a dog or not.

Our study also had some limitations. Firstly, the cross‐sectional design precluded the assessment of causal links; hence, longitudinal studies are needed to reinforce the level of evidence. Secondly, the sample size (especially the number of the dog owners) was relatively small and our recruitment based on users of the healthcare system may limits the representativeness of the sample. Lastly, the study was conducted during and after the COVID pandemic, which might have modified the reporting of loneliness and social isolation. However, we observed similar results over the years of our study (2020–2023).

The results of this study should be interpreted with caution, in view of the small number of participants in each group. It would have been interesting to include cat owners as well as dog owners. Although the body of research on cats and loneliness is less extensive than that on dogs, and the findings are somewhat contradictory, the results of some studies have suggested that cat ownership is associated with less feelings of loneliness ‐ particularly among older adults [15, 33, 43, 44]. Although cats do not encourage mobility outside the home, they impose fewer constraints on older adults and still provide loyal companionship.

There is growing interest in “emotional robots” capable of interacting with people in a more natural, intuitive and empathetic manner, thanks to advances in artificial intelligence. Despite the emergence of these hi‐tech developments, we believe that it is important to continue to explore low‐tech solutions (such as dogs) and thus to maintain a balance between technology and living beings. Learning to further develop this bond in support of older adults while ensuring the pet's well‐being appears to be a promising avenue that complements robot‐based approaches.

## 5 Conclusion

Dog‐owning older people were more likely to report a lower level of loneliness than their non‐owner peers. This association was driven mainly by a lower emotional loneliness subscore. The main reason for having a dog was companionship, followed by social interactions and physical activities. Few adverse events (like falls or bites) were reported.

## Funding

This work was supported by Fondation MUTAC (Fondation de l’Avenir).

## Conflicts of Interest

The authors declare no conflicts of interest.

## Supporting information


Supporting Information S1

## Data Availability

The data that support the findings of this study are available on request from the corresponding author. The data are not publicly available due to privacy or ethical restrictions.
