# Supplementary material for: Dog Companionship and Loneliness in Community‐Dwelling Older Adults: A Multicentre, Cross‐Sectional Study
Source: Int J Geriatr Psychiatry. 2026 May 2;41:e70216. doi: 10.1002/gps.70216 (PMC13135383; doi:10.1002/gps.70216)
Supplement: Supplementary file 1 — Supporting Information S1 [file GPS-41-e70216-s001.docx]

**Appendix 1. De Jong Gierveld Loneliness Scale (11-item version)**

The De Jong Gierveld Loneliness Scale is a validated instrument designed to measure overall, emotional, and social loneliness. It consists of 11 items: 6 negatively worded (indicating loneliness) and 5 positively worded (indicating belongingness).

| Item No. | Statement | Dimension | Response categories** | Coding* |
| --- | --- | --- | --- | --- |
| 1 | I experience a general sense of emptiness. | Emotional loneliness | Yes / More or less / No | Yes, More or less → Lonely; No → Not lonely |
| 2 | I miss having people around. | Emotional loneliness | Yes / More or less / No | Yes, More or less → Lonely; No → Not lonely |
| 3 | I often feel rejected. | Emotional loneliness | Yes / More or less / No | Yes, More or less → Lonely; No → Not lonely |
| 4 | I feel isolated from others. | Emotional loneliness | Yes / More or less / No | Yes, More or less → Lonely; No → Not lonely |
| 5 | There are many people I can trust completely. | Emotional loneliness | Yes / More or less / No | Yes → Not lonely; More or less, No → Lonely |
| 6 | There are plenty of people I can rely on when I have problems. | Emotional loneliness | Yes / More or less / No | Yes → Not lonely; More or less, No → Lonely |
| 7 | There are enough people I feel close to. | Social loneliness | Yes / More or less / No | Yes → Not lonely; More or less, No → Lonely |
| 8 | I can call on my friends whenever I need them. | Social loneliness | Yes / More or less / No | Yes → Not lonely; More or less, No → Lonely |
| 9 | There are many people I can count on completely. | Social loneliness | Yes / More or less / No | Yes → Not lonely; More or less, No → Lonely |
| 10 | I can find companionship when I want it. | Social loneliness | Yes / More or less / No | Yes → Not lonely; More or less, No → Lonely |
| 11 | There are enough people I can rely on in case of need. | Social loneliness | Yes / More or less / No | Yes → Not lonely; More or less, No → Lonely |

*Coding follows the validated dichotomization procedure (De Jong Gierveld & Van Tilburg, 2010).
**Original response scale: “Yes!”, “Yes”, “More or less”, “No”, “No!” → collapsed into Yes / More or less / No.
Total score ranges from 0 (not lonely) to 11 (extremely lonely). Subscales: Emotional loneliness (6 items) and Social loneliness (5 items).

Reference:
De Jong Gierveld J, Van Tilburg TG. The De Jong Gierveld short scales for emotional and social loneliness: tested on data from 7 countries in the UN Generations and Gender Surveys. Eur J Ageing. 2010;7(2):121–130.

**Appendix 2: Social Network Index – 4-Item Short Version**

1. Do you share your life with someone living in the same household as you?

- Yes
- No

1. Do you belong to a social group (association, club, religious group, etc.)?

- Yes
- No

1. How many people do you trust and can rely on?

- >=8
- 4–7
- 0–3

1. How many direct contacts per month do you have with your family or friends, at your home or theirs?

- Many (≥4)
- Moderate (3)
- Few (2)
- Very few or none (0–1)

**Appendix 3. Baseline characteristics of participants, as a function of dog ownership**

|  |  | **Dog ownership** | |  |
| --- | --- | --- | --- | --- |
|  | **Total** | **No** | **Yes** | **p-value*** |
|  | N=160 | N=113 | N=47 |  |
|  | N(%) | N(%) | N(%) |  |
| **Age, years, median [interquartile range]** | 82.00 [77.50-87.00] | 82.00 [78.00-88.00] | 81.00 [77.00-84.00] | **0.046** |
| **Female** | 116 (72.50) | 83 (73.45) | 33 (70.21) | 0.676 |
| **Educational level (MD= 27/16.9%)**** |  |  |  |  |
| No degree/Don't know | 18 (13.53) | 12 (13.04) | 6 (14.63) | 0.053 |
| Primary school certificate | 11 (8.27) | 3 (3.26) | 8 (19.51) |  |
| GCSEs under grade C | 18 (13.53) | 11 (11.96) | 7 (17.07) |  |
| NVQ, BETC or GNVQ | 35 (26.32) | 25 (27.17) | 10 (24.39) |  |
| BETC Higher National Diploma, BA or BS | 20 (15.04) | 15 (16.30) | 5 (12.20) |  |
| MA or MS | 16 (12.03) | 13 (14.13) | 3 (7.32) |  |
| Other | 15 (11.28) | 13 (14.13) | 2 (4.88) |  |
| **Former profession (26/16.3%)** |  |  |  |  |
| Craftsmen, shopkeepers and company directors | 10 (7.46) | 7 (7.53) | 3 (7.32) | 0.442 |
| Executives and higher intellectual professions | 20 (14.93) | 14 (15.05) | 6 (14.63) |  |
| Intermediate professions | 19 (14.18) | 12 (12.90) | 7 (17.07) |  |
| Employees | 44 (32.84) | 33 (35.48) | 11 (26.83) |  |
| Workers | 4 (2.99) | 1 (1.08) | 3 (7.32) |  |
| Not working or at home | 9 (6.72) | 5 (5.38) | 4 (9.76) |  |
| Other | 28 (20.90) | 21 (22.58) | 7 (17.07) |  |
| **Inclusion at a hospital vs a primary care centre** | 105 (65.63) | 87 (76.99) | 18 (38.30) | **0.000** |
| **Living alone** | 79 (49.38) | 57 (50.44) | 22 (46.81) | 0.675 |
| **Living space (24/15.0%)** |  |  |  |  |
| Personal home | 132 (97.06) | 91 (96.81) | 41 (97.62) | 1.000 |
| With family member/third party | 1 (0.74) | 1 (1.06) | 0 (0.00) |  |
| Residential home/residence for older adults | 3 (2.21) | 2 (2.13) | 1 (2.38) |  |
| **Distance from a green space (24/15.0%)** |  |  |  |  |
| At home (garden) | 61 (44.85) | 32 (34.04) | 29 (69.05) | **0.001** |
| Between 0 m and 200 m | 39 (28.68) | 30 (31.91) | 9 (21.43) |  |
| Between 200 m and 500 m | 18 (13.24) | 16 (17.02) | 2 (4.76) |  |
| More than 500m | 18 (13.24) | 16 (17.02) | 2 (4.76) |  |
| **Current smoking (2/1.3%)** |  |  |  |  |
| Non-smoker | 109 (68.99) | 82 (72.57) | 27 (60.00) | 0.301 |
| Active smoker | 13 (8.23) | 8 (7.08) | 5 (11.11) |  |
| Former smoker | 36 (22.78) | 23 (20.35) | 13 (28.89) |  |
| **Current general condition (6/3.8%)** |  |  |  |  |
| Very good | 39 (25.32) | 28 (25.23) | 11 (25.58) | 0.878 |
| Good | 83 (53.90) | 61 (54.95) | 22 (51.16) |  |
| Fair | 32 (20.78) | 22 (19.82) | 10 (23.26) |  |
| **BMI, kg/m², median [interquartile range] (3/1.9%)** | 24.34 [21.46-27.64] | 23.88 [21.39-27.64] | 24.70 [21.71-27.64] | 0.471 |
| **Number of medications taken daily, median [interquartile range] (17/10.6%)** | 5.00 [2.00-7.00] | 4.00 [2.00-6.00] | 6.00 [3.00-8.00] | **0.032** |
| **Number of illnesses requiring regular treatment, median [interquartile range] (17/10.6%)** | 3.00 [2.00-3.00] | 2.50 [2.00-3.00] | 3.00 [2.00-4.00] | 0.188 |
| **Overall CIRS-G score, median [interquartile range] (7/4.4%)** | 20.00 [17.00-21.00] | 19.00 [17.00-21.00] | 20.00 [18.00-22.00] | 0.079 |
| **IADL score, median [interquartile range]** | 8.00 [7.00-8.00] | 8.00 [7.00-8.00] | 7.00 [5.00-8.00] | **0.001** |
| **ADL score, median [interquartile range]** | 6.00 [5.50-6.00] | 6.00 [5.50-6.00] | 6.00 [5.50-6.00] | 0.797 |
| **Marshall score, median [interquartile range] (1/0.6%)** | 1.00 [0.00-2.00] | 1.00 [0.00-2.00] | 0.50 [0.00-4.00] | 0.733 |
| **Frail/very frail person (13/8.1%)** | 46 (31.29) | 30 (29.13) | 16 (36.36) | 0.386 |
| **Has a main carer** | 45 (33.33) | 34 (36.17) | 11 (26.83) | 0.290 |
| **No daily physical activity (1/0.6%)** | 43 (27.04) | 25 (22.12) | 18 (39.13) | **0.029** |
| **Number of minutes walked/day, median- [interquartile range] (46/28.8%)** | 42.50 [30.00-60.00] | 30.00 [25.00-60.00] | 45.00 [30.00-60.00] | 0.339 |
| **Number of falls, median [interquartile range] (2/1.3%)** | 1.00 [1.00-2.00] | 1.00 [1.00-2.00] | 1.00 [1.00-3.00] | 0.386 |
| **History of falls in the last 6 months (1/0.6%)** | 56 (35.22) | 39 (34.82) | 17 (36.17) | 0.871 |
| **Fall with complications (4/7.1%)** | 15 (28.85) | 13 (35.14) | 2 (13.33) | 0.179 |
| **Post-fall syndrome (3/5.4%)** | 11 (20.75) | 8 (22.22) | 3 (17.65) | 1.000 |
| **Slightly impaired cognitive function (2/1.3%)** | 24 (15.19) | 17 (15.18) | 7 (15.22) | 0.995 |
| **Abnormal GDS score (short-form) (4/2.5%)** | 72 (46.15) | 50 (44.64) | 22 (50.00) | 0.546 |
| **Current anxiety disorder (2/1.3%)** | 68 (43.04) | 45 (40.54) | 23 (48.94) | 0.330 |
| **Current depressive syndrome** | 40 (25.00) | 27 (23.89) | 13 (27.66) | 0.616 |
| **Fatigue** |  |  |  |  |
| Never | 32 (20.00) | 22 (19.47) | 10 (21.28) | 0.950 |
| Sometimes | 67 (41.88) | 49 (43.36) | 18 (38.30) |  |
| Often | 26 (16.25) | 18 (15.93) | 8 (17.02) |  |
| Very often/constantly | 35 (21.88) | 24 (21.24) | 11 (23.40) |  |
| **Sleep problems** |  |  |  |  |
| Never | 43 (26.88) | 38 (33.63) | 5 (10.64) | **0.016** |
| Sometimes | 59 (36.88) | 35 (30.97) | 24 (51.06) |  |
| Often | 24 (15.00) | 16 (14.16) | 8 (17.02) |  |
| Very often/constantly | 34 (21.25) | 24 (21.24) | 10 (21.28) |  |

Missing: (/%) indicates the number and % of missing data. Abbreviations: ADL, activities of daily living; BA, Bachelor of Arts; BS, Bachelor of Science; BTEC, Business and Technology Education Council; BMI, body mass index; CIRS-G, Cumulative Illness Rating Scale-Geriatric, GCSE, General Certificate of Secondary Education; GDS, Geriatric Depression Scale; GNVQ, General National Vocational Qualification; IADL, instrumental activities of daily living; MA, Master of Arts; MD, missing data; MS, Master of Science; NVQ, National Vocational Qualification. * P value in a Student’s test, Mann-Whitney test, chi-squared test, or Fisher’s test, as appropriate.

** These are the UK equivalents of the French educational levels observed in the present study

**Appendix 4: Table of Individual characteristics, relationship with dogs, and dog characteristics**

|  | **Total** |
| --- | --- |
|  | N=160 |
|  | N (%) |
| **Dog owners (N=47), 37 respondents** |  |
| **Reasons cited for owning a dog*** |  |
| Companionship | 30 (81.1) |
| Go out/do physical activity | 13 (35.1) |
| Hearing or visual aid | 0 (0.0) |
| Hunting | 1 (2.7) |
| Offered by a third party or found abandoned | 18 (48.7) |
| Security/guard | 0 (0.0) |
| **Age of the dog** | 6 [3.5-9] |
| **Sex of the dog, female** | 19 (51.4) |
| **Length of relationship with the dog** | 4 [3-6.6] |
| **Emotional support provided by the dog** | 10 [9-10] |
| **Animal behaviour with other dogs** |  |
| Solitary | 3 (8.1) |
| Sociable | 27 (73.0) |
| Aggressive | 7 (18.9) |
| **Animal behaviour with other individuals** |  |
| Solitary | 3 (8.1) |
| Sociable | 33 (89.2) |
| Aggressive | 1 (2.7) |
| **Expanding the network of close friends thanks to the dog** | 13 (35.1) |
| **Walk the dog** | 25 (67.6) |
| **Frequency of walks** |  |
| A few times a week | 4 (16.0) |
| Once a day | 8 (32.0) |
| Twice a day | 6 (24.0) |
| More than twice a day | 7 (28.0) |
| **Incident involving the dog*** |  |
| Bite | 2 (5.4) |
| Fall | 2 (5.4) |
| Degradation of the home | 10 (27.0) |
| **Individuals without a dog (N=113)** |  |
| **The reasons for not owning a dog*** |  |
| Responsibilities deemed to be too great (n=84) | 52 (60.5) |
| Old age (n=84) | 21 (25.0) |
| Unsuitable accommodation (n=86) | 14 (16.3) |
| State of health (n=85) | 8 (9.4) |
| Financing cost (n=84) | 2 (2.4) |
| Fear of animals (n=85) | 0 (0.0) |
| Grief due to the death of a previous pet (n=86) | 8 (9.3) |
| Does not like dogs (n=85) | 5 (5.9) |
| Other constraints (n=85)** | 18 (21.2) |
| *non-mutually exclusive categories  ** Housing refusals, travel, neighbourhood |  |
